# Integrative genomic analysis reveals DHX58 as a key player in gastric cancer

**DOI:** 10.1371/journal.pone.0341230

**Published:** 2026-01-22

**Authors:** Yan Li, Liren Zhang, Zhi Wen, Haixia Wang, Kunming Zhang, Buzai Wang, Jinfan Li, Huiqing Kang, Yanjun Gao, Zhi Yu, Yongzhe Du

**Affiliations:** 1 Department of Gastroenterology, Wuhai People’s Hospital, Wuhai, China; 2 Dispensary for Western Medicine, Wuhai People’s Hospital, Wuhai, China; 3 Department of Medical Imaging, Wuhai People’s Hospital, Wuhai, China; 4 Department of Pathology, Wuhai People’s Hospital, Wuhai, China; CRCL: Centre de Recherche en Cancerologie de Lyon, FRANCE

## Abstract

Gastric cancer (GC) is a major global health burden with limited treatment options. Identifying the molecular mechanisms underlying GC progression is critical for developing novel therapeutic strategies. We integrated whole-genome bisulfite sequencing and bulk RNA sequencing to identify hub genes involved in GC. Functional annotations were performed using Gene Ontology and Kyoto Encyclopedia of Genes and Genomes enrichment analyses, supplemented by data from The Cancer Genome Atlas. Key findings were validated using western blotting, immunohistochemistry, quantitative real-time PCR, and methylation-specific PCR. DExH-box helicase 58 (*DHX58*) was identified as a hypomethylated, highly expressed hub gene in GC. Mechanistically, *DHX58* expression is regulated by the transcription factor CCAAT enhancer–binding protein α. Immune profiling further implicated *DHX58* in the development of resistance to GC immunotherapy. Our study revealed that *DHX58* hypomethylation drives its overexpression in GC, making it a promising therapeutic target. These findings offer new insights into the pathogenesis of GC and suggest potential immunotherapeutic approaches.

## Introduction

Gastric cancer (GC) is the fifth most commonly occurring malignancy and the fourth leading cause of cancer-related mortality worldwide [1]. Despite decreasing incidence in some regions, the 5-year survival rate for advanced GC remains below 30%, underscoring the urgent need for novel therapeutic targets [2]. Accumulating evidence implicates epigenetic dysregulation as a critical driver of gastric carcinogenesis [3], with DNA hypomethylation emerging as a key oncogenic mechanism that activates silenced genomic regions [4]. Although genome-wide hypomethylation is a recognized hallmark of cancer [5], its specific impact on immune-related genes in GC remains poorly characterized.

DExH-box helicase 58 (*DHX58*, also known as LGP2) is a cytoplasmic RNA helicase that is a key component of the retinoic acid–inducible gene-I (RIG-I)-like receptor (RLR) family, the members of which are critical modulators of antiviral innate immunity. Unlike RIG-I and melanoma differentiation-associated gene 5 (MDA5), *DHX58* lacks caspase activation and recruitment domains, which enables it to fine-tune RLR signaling through a dual mechanism: it amplifies MDA5-mediated interferon (IFN) responses while suppressing RIG-I-dependent pathways by competitively binding to viral RNA and regulating RIG-I ubiquitination [6,7]. This duality underpins its context-dependent roles in inflammation and cancer. However, the regulation of *DHX58* through promoter methylation and its immunomodulatory roles in GC are currently unknown.

To address this knowledge gap, we combined multiomics analyses with experimental validation to verify the status of *DHX58* as a methylation-driven hub gene. This study highlights the role of hypomethylation in *DHX58* overexpression, the CCAAT enhancer–binding protein α (CEBPα)-dependent mechanism controlling *DHX58* transcription, the resulting reprogramming of the immune microenvironment, and the clinical significance of *DHX58* as a predictor of immunotherapy resistance.

## Materials and methods

### Construction of a rat GC model

Five-week-old male Wistar rats (130 ± 10 g each) received N-methyl-N′-nitro-N-nitrosoguanidine (MNNG, 120 μg/L in drinking water kept at 4°C in light-protected bottles, replaced every 1–2 days) and 0.05% ranitidine feed. The rats were starved on Tuesdays/Thursdays from 20:00–08:00 to simulate feeding disorders, followed by 2% sodium salicylate gavage (5 mL/kg) to mimic damage caused by nonsteroidal anti-inflammatory drugs. The MNNG/ranitidine feed was started in week 1; full interventions (fasting/salicylate) were initiated in week 2. The GC model was established after 28 weeks. Sequencing was performed using four control rats and four GC model rats (euthanized after 28 weeks), and the collected gastric tissues were split for histological and DNA methylation analyses. All procedures followed the National Institutes of Health guidelines (2011) and were granted institutional approval (approval number: whsrmyyky20210601).

Euthanasia was performed on rats by slow intraperitoneal injection of pentobarbital sodium using a 25G needle. The 3R principles were followed to protect animal welfare, optimize the breeding environment, provide sterile food and water, enrich the environment, and conduct regular cleaning. The rat gastric cancer model was independently and repeatedly constructed three times, and randomization and blinding were maintained during tissue sampling and sequencing.

### RNA, DNA, and protein extraction

RNA, DNA, and proteins were isolated from gastric tissue samples using TRIzol reagent (Wuxi Zhanwang Chemical Reagent Co., Ltd.), the Bio-Tek DNA Kit (Omega), and the bicinchoninic acid (BCA) protein assay kit (Thermo Fisher Scientific), respectively. For each group, aliquots derived from the same gastric tissue sample were randomly selected for DNA and RNA extraction and subsequent sequencing analyses.

### Whole-genome bisulfite sequencing (WGBS)

Genomic DNA was isolated from the stomach tissues of rats using the AllPrep DNA/RNA Mini Kit (Qiagen). WGBS was performed as previously described [8]. Briefly, 1 µg of genomic DNA was sonicated to generate approximately 300-bp-long fragments using the M220 ultrasonicator (Covaris). Sequencing libraries were constructed using the NEBNext Ultra II DNA Library Prep Kit, treated with bisulfite using the Imprint DNA Modification Kit, and ligated to Illumina adapters. Libraries were size-selected (300–600 bp) using the Pippin Prep System, amplified using Pfu-Turbo Cx Hotstart DNA polymerase, and sequenced on the Illumina HiSeq 2000 platform to generate 100-bp paired-end reads. Three biological replicates per group were analyzed.

### Whole-messenger transcriptome sequencing

Total RNA was isolated using TRIzol reagent. RNA integrity was assessed using the NanoDrop One spectrophotometer, Agilent 2100 Bioanalyzer, and Fragment Analyzer (Advanced Analytical Technologies). The isolated RNA was quantified using the Qubit RNA HS Assay Kit (Thermo Fisher Scientific), and the concentration of each RNA sample was normalized to 100 ng/μL. Samples with RNA integrity number ≥ 6 were selected for library preparation. Libraries were constructed using the VAHTS Universal V8 RNA-seq Kit (Illumina) through mRNA enrichment using oligo-dT beads, fragmentation, cDNA synthesis, adapter ligation, and PCR amplification. Paired-end sequencing (126-bp reads) was performed on an Illumina HiSeq 2500v4 system (≥12.5 Gb/sample; ~ 50 million read pairs). Data were processed using the RTA v1.18.64 and Bcl2fastq v1.8.4 tools, with reads deposited in Sanger-compressed FASTQ format.

### Western blotting

Tissues were lysed in RIPA buffer (Beyotime Biotechnology) containing 1 mM phenylmethylsulfonyl fluoride. Protein concentrations were determined using the BCA assay (Beyotime Biotechnology). Samples containing equal amounts of protein (20 μg) were separated via electrophoresis using 10% sodium dodecyl sulfate–polyacrylamide gels, and the resolved proteins were transferred to polyvinylidene fluoride membranes. The membranes were blocked using 5% nonfat milk in Tris-buffered saline containing 0.05% Tween-20 (TBST), probed overnight at 4°C with primary antibodies, incubated for 1 h at room temperature with species-matched horseradish peroxidase (HRP)-conjugated secondary antibodies, and washed five times using TBST. Protein bands were detected using enhanced chemiluminescence on the ImageQuant LAS 4000 mini digital imaging system (GE Healthcare). Three independent experiments were performed.

### Immunohistochemistry

Rat gastric sections (4-μm-thick) were deparaffinized, rehydrated, treated with 3% H_2_O_2_ for 10 min to quench endogenous peroxidase, incubated with 5% bovine serum albumin for 30 min at room temperature to block nonspecific binding, probed with primary antibodies overnight at 4°C, incubated with HRP-conjugated secondary antibodies, washed, and finally subjected to 3,3′-diaminobenzidine development and hematoxylin counterstaining.

### RNA extraction and quantitative real-time PCR

Total RNA was extracted using TRIzol reagent (Invitrogen). cDNA was synthesized using M-MLV reverse transcriptase (Invitrogen). To quantify gene expression, quantitative real-time PCR was performed using SYBR Green Master Mix (TaKaRa) on an ABI 7900 system (Applied Biosystems). GAPDH was used as an endogenous control. Primer amplification efficiency (90%–110%) was validated using standard curves. Relative gene expression was calculated using the 2 ⁻ ^ΔΔCt^ method. The primers used were as follows: *DHX58*-RAT-F, CTGCTCTGGAGGGCAAGAAC; *DHX58*-RAT-R, TTCAGCGCCAGATGCAGTAA; rat actin beta-RT-F, CACCCGCGAGTACAACCTTC; rat actin beta-RT-R, CCCATACCCACCATCACACC.

### Methylation-specific PCR (MS-PCR)

The DNA methylation status of *DHX58* was determined using MS-PCR. DNA was extracted from gastric tissues using DNAzol™ (Thermo Fisher), subjected to bisulfite conversion using the EZ DNA Methylation-Gold Kit (Zymo Research), and amplified using methylated/unmethylated allele-specific primers. The PCR cycling conditions were as follows: initial denaturation at 95°C/5 min; 40 cycles at 95°C/20 s, 60°C/20 s, 72°C/30 s; and final extension at 72°C/5 min. The products (8 μL) were electrophoresed on 3% agarose gels, stained with SYBR Gold (Thermo Fisher), and imaged under ultraviolet light. The following primers were used: left M primer, TTTTATCGAGTTATGTAGGTTGAGO; right M primer, TAAACAACAATATAAAACGAACCGA; left U primer, TTATTGAGTTATGTAGGTTGAGTGT; right U primer, AAACAACAATATAAAACAAACCAAA.

### Bioinformatics analysis of immune infiltration and therapeutic efficacy

GC RNA sequencing data (STAR-counts) and clinical records were obtained from The Cancer Genome Atlas (TCGA) project hosted on the Genomic Data Commons portal. The data were subjected to log_2_(TPM + 1) transformation and sample filtration (N = 375 with matched molecular/clinical data). Spearman’s correlation analysis was used for non-normally distributed variables, and immunotherapy response prediction was performed using the TIDE algorithm. Statistical procedures were implemented in R software, v4.0.3.

### Statistical analysis

Data are expressed as means ± standard errors of the mean. Differences among groups were analyzed using one-way analysis of variance with Tukey’s and least significant difference tests. P < 0.05 was considered to denote a significant difference. All analyses were performed using GraphPad Prism 9.0 software.

## Results

### Analysis of the global methylation status in rats with GC

**Fig 1:**
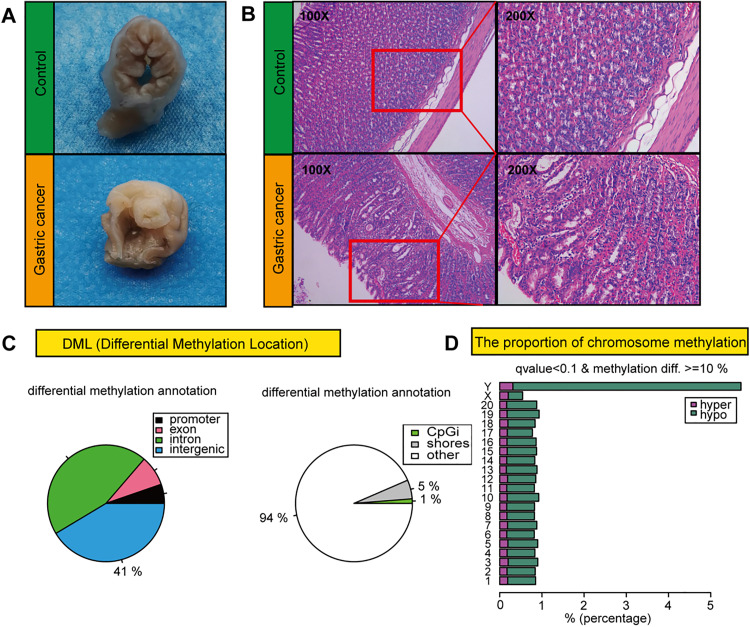
Analysis of methylation in rats with GC. **(A)** Images of gastric tissues from the control and GC groups. **(B)** HE staining of gastric tissues from the control and GC groups. **(C)** Pie charts show the genomic locations of the identified DMRs (promoter, 5′ UTR, exon, intron, 3′ UTR, intergenic). **(D)** Proportion of high/low methylation sites in each chromosome. GC, gastric cancer; HE, hematoxylin and eosin; DMRs, differentially methylated regions; UTR, untranslated region.

To investigate the changes in methylation and the transcriptome between normal rats and rats with GC, we first successfully established a rat GC model (Fig 1A). Immunohistochemical analysis was used to verify the histopathological authenticity of both GC tissues and normal gastric tissues (Fig 1B).

Through comparative analysis of GC and normal gastric tissues, we identified over 1000 differentially methylated regions (DMRs), most of which were located in intergenic and intronic regions (Fig 1C). Furthermore, chromosomal distribution analysis demonstrated an increased density of hypermethylated sites in GC tissues compared with control tissues (Fig 1D).

**Fig 2:**
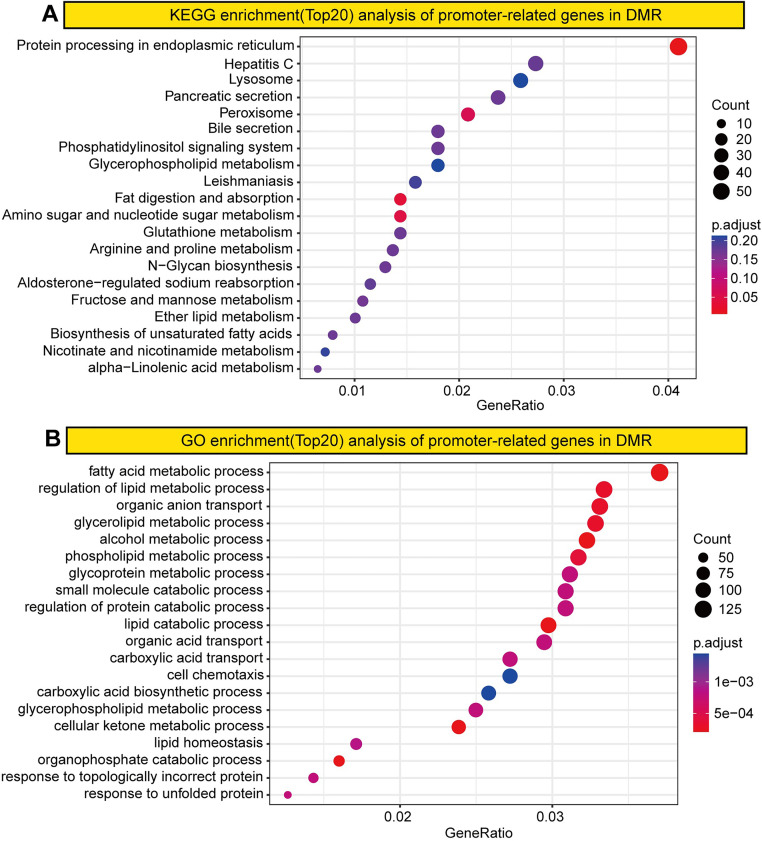
Enrichment analysis of DMRs. **(A)** KEGG enrichment. **(B)** GO enrichment.

**Fig 3:**
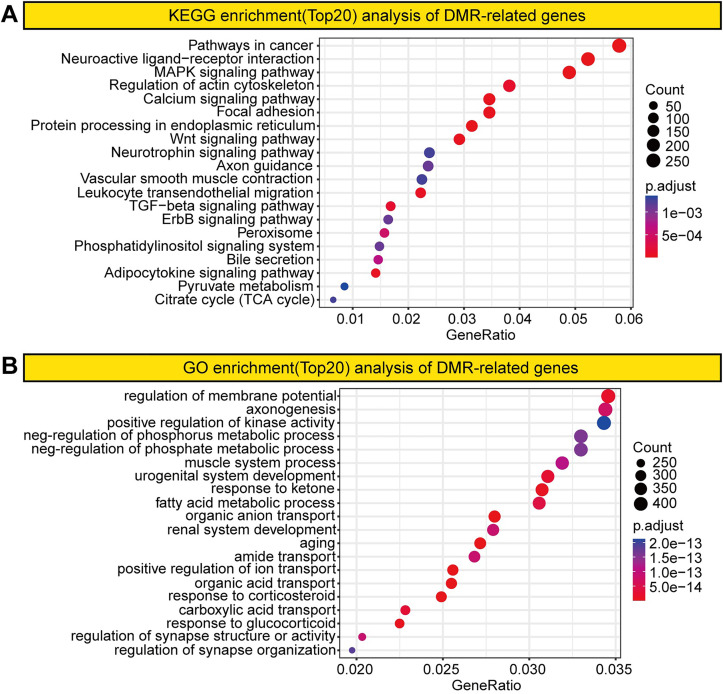
Enrichment analysis of promoter-related genes in DMRs. **(A)** KEGG enrichment. **(B)** GO enrichment.

To investigate DNA methylation changes in GC, we analyzed the genes associated with the identified DMRs. Gene Ontology (GO) and Kyoto Encyclopedia of Genes and Genomes (KEGG) analyses revealed GC-specific enrichment in pathways related to membrane potential regulation, cancer progression, neuroactive ligand–receptor interactions, and mitogen-activated protein kinase signaling, which may be linked to genome-wide hypomethylation (Fig 2). Additionally, GO and KEGG analyses revealed significant enrichment in processes such as protein processing in the endoplasmic reticulum, hepatitis C infection, lysosome function, fatty acid metabolism, lipid metabolism regulation, and organic anion transport (Fig 3).

### Transcriptomic analysis of rats with GC

**Fig 4:**
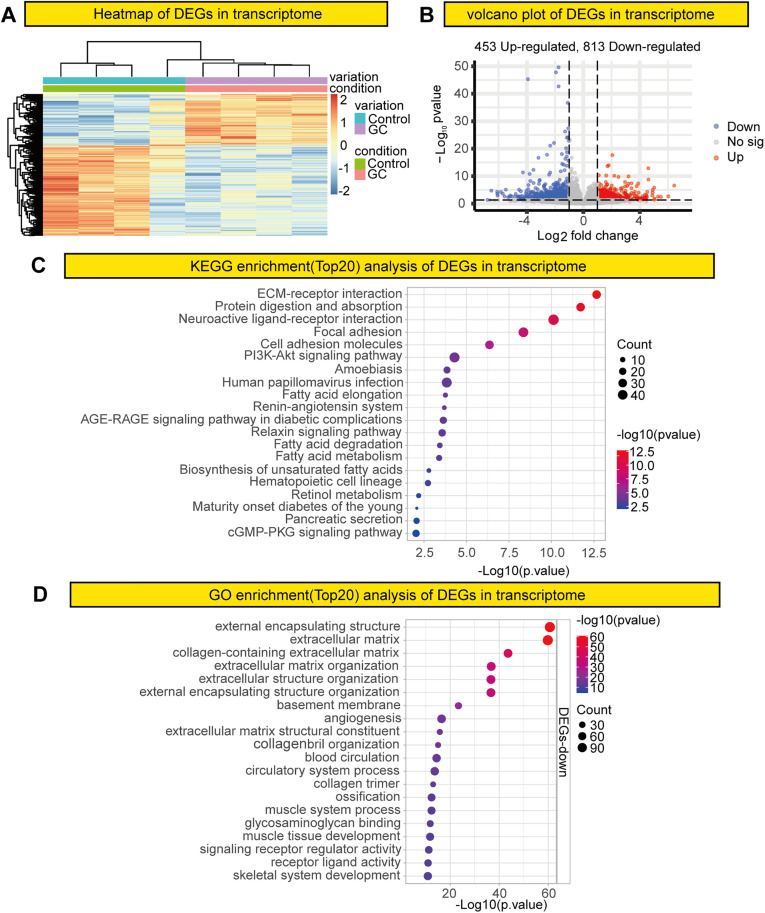
Transcriptome sequencing analysis of rats with GC. **(A)** Heat map of DEGs. **(B)** Volcano plot of gene expression patterns. **(C)** Dot plot of GO enrichment. **(D)** Dot plot of KEGG enrichment. DEGs, differentially expressed genes; GO, Gene Ontology; KEGG, Kyoto Encyclopedia of Genes and Genomes.

A hierarchical clustering algorithm was employed to perform cluster analysis on the significantly differentially expressed genes (DEGs) identified between the control and GC groups, and the results were visualized in the form of a heat map. The heat map illustrates both DEGs between the control and GC groups as well as genes with similar expression profiles (Fig 4A). Given the changes observed in the methylome of GC rats, we aimed to investigate the potential effects of these changes on the transcriptome. Transcriptomic analysis revealed that GC rats exhibited differential expression of 1266 genes compared with control rats, respectively (Fig 4B).

We used the ClusterProfiler software package to perform GO and KEGG functional enrichment analyses of the DEGs identified through RNA sequencing. Pathway analysis was used to identify key pathways associated with the DEGs in GC, revealing that the pathways most affected were external encapsulation structure, extracellular matrix, extracellular matrix–receptor interaction, protein digestion and absorption, and neuroactive ligand–receptor interaction (Fig 4C, D).

### Hub gene screening in GC

**Fig 5:**
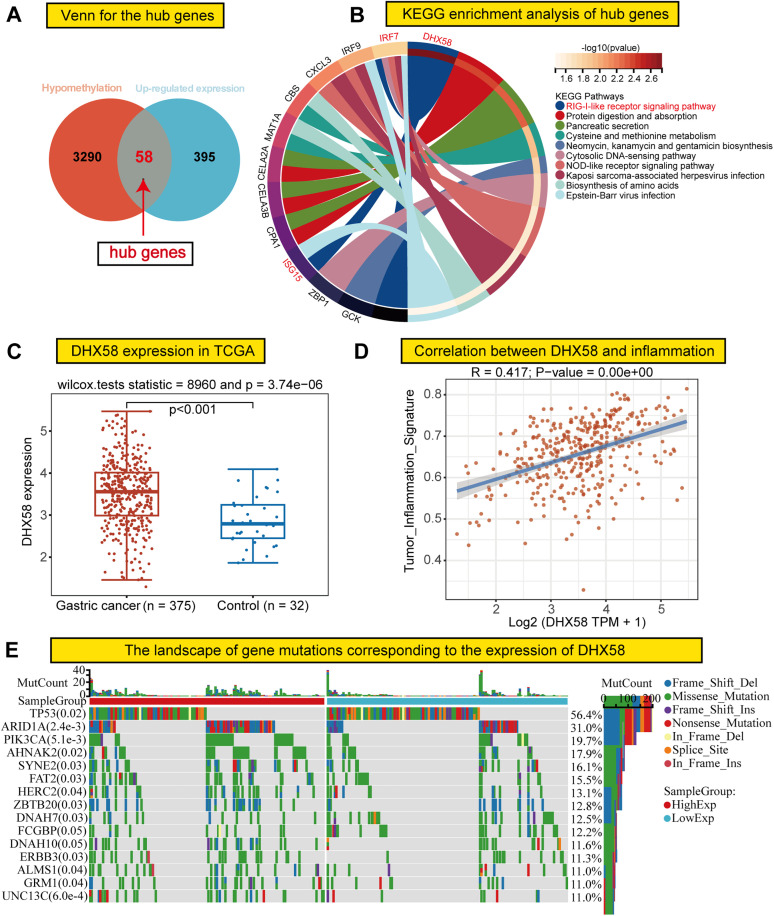
Screening of key genes and related analysis. **(A)** Venn diagram of hypomethylated and highly expressed genes. **(B)** KEGG circle diagram analysis of key pathways and core genes related to hub genes. **(C)** mRNA expression level of *DHX58* in GC and adjacent tissues from the TCGA database. **(D)** Spearman correlation analysis of *DHX58* and inflammatory pathways. GC, GC-adjacent tissue; TCGA, TCGA. **(E)** Corresponding gene mutation profiles of the two groups with high and low *DHX58* expression levels. *DHX58*, DExH-box helicase 58; TCGA, The Cancer Genome Atlas.

To investigate the potential relationships between GC-induced changes in DNA methylation and gene expression, a comprehensive analysis of methylation and transcriptomic profiles was performed. A Venn diagram demonstrated that 58 genes exhibited decreased methylation levels alongside increased expression in the GC group (Fig 5A). Furthermore, KEGG enrichment analysis revealed that the aforementioned hub genes were significantly associated with the RIG-I-like receptor signaling pathway, with *DHX58* being the most highly weighted gene (Fig 5B). Therefore, we chose to focus on *DHX58* as a key hub gene in GC.

In a supplementary analysis, *DHX58* was found to be significantly overexpressed in GC samples from the TCGA database (Fig 5C). Moreover, our findings suggest that *DHX58* is closely associated with the inflammatory pathway (Fig 5D). Additionally, we conducted an in-depth analysis of the overall gene mutation profiles between the high- and low-expression groups stratified by *DHX58* expression. The results revealed that TP53 mutations were the most prominent alteration (Fig 5E).

### Analysis of immune infiltration and therapeutic efficacy of DHX58

**Fig 6:**
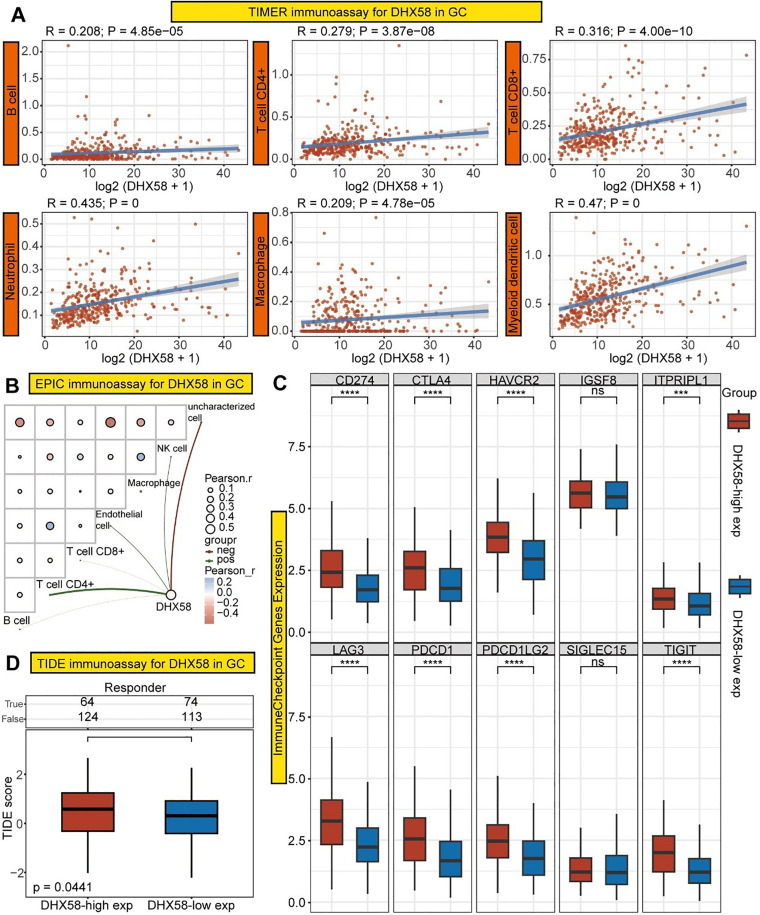
Immune-related analysis of *DHX58.* **(A)** TIMER analysis of the correlation between *DHX58* expression and six types of immune cells. **(B)** EPIC analysis of the correlation between *DHX58* expression and six types of immune cells. **(C)** Relationship between *DHX58* expression and 10 immune checkpoint-related molecules. **(D)** TIDE score of *DHX58* was used to analyze its ability to predict the efficacy of immunotherapy.

To further elucidate the critical role of *DHX58* in GC, we conducted an immunological analysis to examine the correlation between *DHX58* expression and six types of immune cells. The results of TIMER immune analysis revealed a significant association between *DHX58* expression and the counts of B cells, T cells, neutrophils, macrophages, and dendritic cells. These findings imply that *DHX58* contributes to carcinogenesis by modulating the immune microenvironment (Fig 6A). EPIC immune analysis corroborated the involvement of *DHX58* in immune regulation (Fig 6B).

Immunotherapy has emerged as a key focus in antitumor research. To elucidate the specific mechanisms through which *DHX58* influences immune regulation, we conducted an analysis targeting 10 key molecules associated with immune checkpoints. The findings revealed a significant correlation between *DHX58* expression and immune checkpoint pathways (Fig 6C). Furthermore, a higher TIDE score in cases of high *DHX58* expression was predictive of a poor response to immunotherapy (Fig 6D).

### Experimental verification of the upstream transcription factors of DHX58

**Fig 7:**
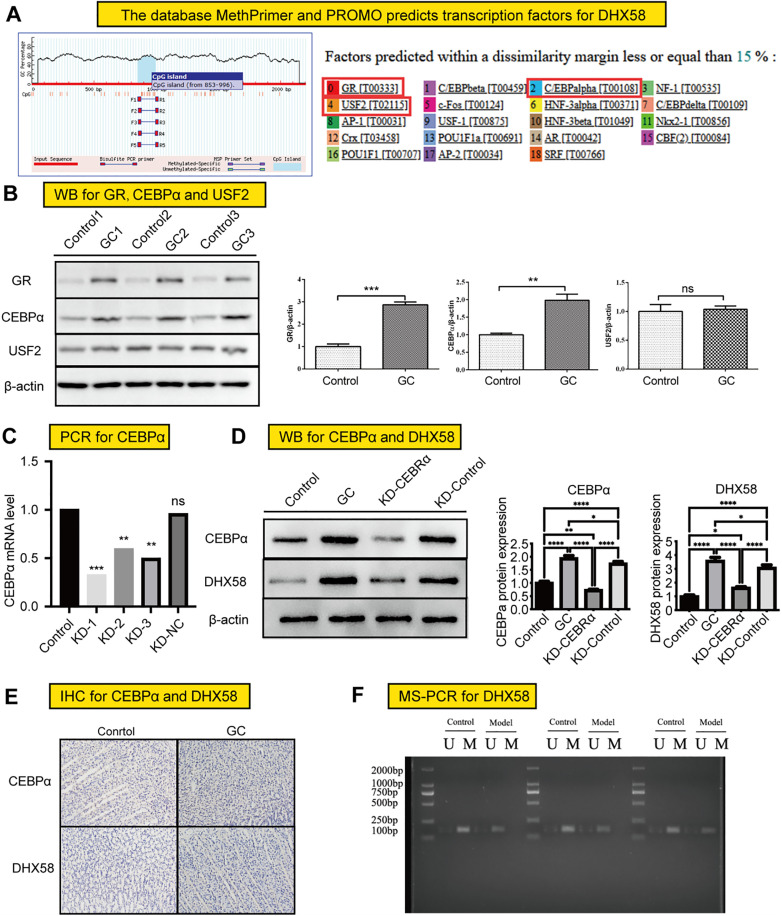
Analysis of upstream transcription factors of *DHX58.* **(A)** Preliminary screening of three transcription factors was conducted based on their binding sites within the CpG island region and the distribution frequency of these binding sites. **(B)** Western blotting was used to detect the expression levels of transcription factors in gastric tissues. **(C)** Knockdown efficiency of CEBPα in AGS cells was detected using PCR. **(D)** Western blotting was used to detect the expression of CEBPα and *DHX58* in AGS cells. **(E)** Detection of the expression of CEBPα and *DHX58* in rat gastric tissues. **(F)** Detection of *DHX58* expression using MS-PCR. AGS, adenocarcinoma gastric stomach; CEBPα, CCAAT enhancer–binding protein α; MS-PCR, methylation-specific PCR.

We investigated the regulatory factors, such as transcription factors, associated with the function of methylation sites. We retrieved the *DHX58* (rat) gene sequence from the National Center for Biotechnology Information database. Given that this is a retrotranscribed gene, the rightmost side of the sequence was designated as the starting point, and the region spanning +100 to −2000 nucleotides was considered as its promoter region. We used the MethPrimer database to predict the distribution of methylated CpG islands within the *DHX58* promoter region (Fig 7A). Subsequently, by integrating the PROMO prediction database, we predicted potential transcription factors for the promoter region sequence. Based on the CpG island region binding sites and the frequency distribution of transcription factor binding sites, three transcription factors were preliminarily screened: glucocorticoid receptor, upstream stimulatory factor 2, and CEBPα (Fig 7B). These methylation-regulated genes and networks may mediate the process of gastric inflammatory cancer transformation.

In this study, western blotting was used to investigate changes in the protein levels of glucocorticoid receptor, upstream stimulatory factor 2, and CEBPα in gastric tissues. Our findings demonstrated that the expression of glucocorticoid receptor and CEBPα was significantly upregulated in GC rats compared with control rats, whereas no significant difference was observed in the expression of upstream stimulatory factor 2 (Fig 7C). Subsequently, we performed knockdown of CEBPα and validated it using PCR (Fig 7D). Western blotting analysis further confirmed that the downregulation of CEBPα resulted in a decrease in the expression level of *DHX58* (Fig 7E), suggesting that CEBPα acts as a transcription factor for *DHX58*. Additionally, we conducted an expression analysis of CEBPα and *DHX58* in rat tumor models, revealing that both CEBPA and *DHX58* were highly expressed in tumors, whereas their expression was markedly lower in normal gastric tissues. Furthermore, MS-PCR was used to detect DNA methylation status, and the results indicated that *DHX58* exhibited hypomethylation in the GC group compared with the control group (Fig 7F).

## Discussion

GC is the fifth most common cancer and the fourth leading cause of cancer-related mortality worldwide [1]. Notably, GC is characterized by a high degree of malignancy, which contributes to its poor prognosis. Overall survival rates remain suboptimal primarily owing to late-stage diagnosis and the presence of metastasis at the time of detection [9,10]. Early screening and accurate diagnosis are critical to mitigate the burden of GC [11]. Although upper gastrointestinal endoscopy remains the gold standard for GC screening, the detection of occult gastric tumors requires further improvements in sensitivity and specificity. Recent studies have explored novel therapeutic strategies for GC based on molecular mechanisms [12].

Epigenetic dysregulation plays a pivotal role in the initiation, progression, and therapeutic management of malignant tumors [13]. Accumulating evidence highlights the strong association between aberrant DNA methylation patterns and GC development [14,15]. Herein, we integrated multiomics approaches, including WGBS, RNA sequencing, and TCGA data, with rigorous experimental validation to identify *DHX58* as a key methylation-driven hub gene in GC. Furthermore, the growing body of research on the diversity of protein modifications reinforces its significance as a promising target and biomarker for advancing cancer prognosis, diagnosis, and therapeutic strategies [16,17].

Previous studies have demonstrated that increased methylation in promoter regions can inhibit gene transcription [18]. Hypomethylation of *DHX58* drives its overexpression in GC, which is associated with increased immune cell infiltration (e.g., B cells, dendritic cells) and upregulation of checkpoint molecules (e.g., programmed cell death protein 1, cytotoxic T-lymphocyte antigen-4). High *DHX58* expression was correlated with poor immunotherapy response, as indicated by elevated TIDE scores. In contrast, in liver cancer, *DHX58* was shown to exhibit tumor-suppressive properties. RNF157-mediated ubiquitination promotes *DHX58* degradation, thereby accelerating tumor proliferation, whereas MATR3 enhances carcinogenesis by inhibiting *DHX58*-dependent IFN-I signaling. This tissue-specific functional divergence may result from interactions with TP53 mutations or differential engagement of the RLR pathway.

We explored two feasible translational directions for DHX58: as a predictive biomarker, its liquid biopsy-detectable methylation status may improve GC immunotherapy response prediction accuracy; as a therapeutic target, inhibiting the CEBPα-DHX58 axis (e.g., via CEBPα small-molecule inhibitors or DHX58 siRNA) may reverse the tumor microenvironment’s immunosuppressive state.

This study has certain limitations. The precise mechanism through which *DHX58* regulates immune cell infiltration requires additional validation, potentially through co-culture assays or other experimental approaches. Furthermore, larger clinical cohorts are essential to robustly confirm the value of *DHX58* as a predictor of immunotherapy resistance.

## Conclusion

We demonstrated that hypomethylation of *DHX58* drives GC progression through CEBPα-mediated transcription and the establishment of an immunosuppressive microenvironment. Targeting this pathway could potentially help in overcoming immunotherapy resistance, thereby providing novel opportunities for precision treatments in patients with GC.

## Supporting information

S1 File Amplification Plot and Melt Curve Plot. (DOCX)

S2 File ACTIN. (TIF)

S3 File CEBPa. (TIF)

S4 File gr. (TIF)

S5 File usf2. (TIF)
