# Supplementary material for: Integrative genomic analysis reveals DHX58 as a key player in gastric cancer
Source: PLoS One. 2026 Jan 22;21(1):e0341230. doi: 10.1371/journal.pone.0341230 (PMC12826466; doi:10.1371/journal.pone.0341230)
Supplement: S1 File — (DOCX) [file pone.0341230.s001.docx]

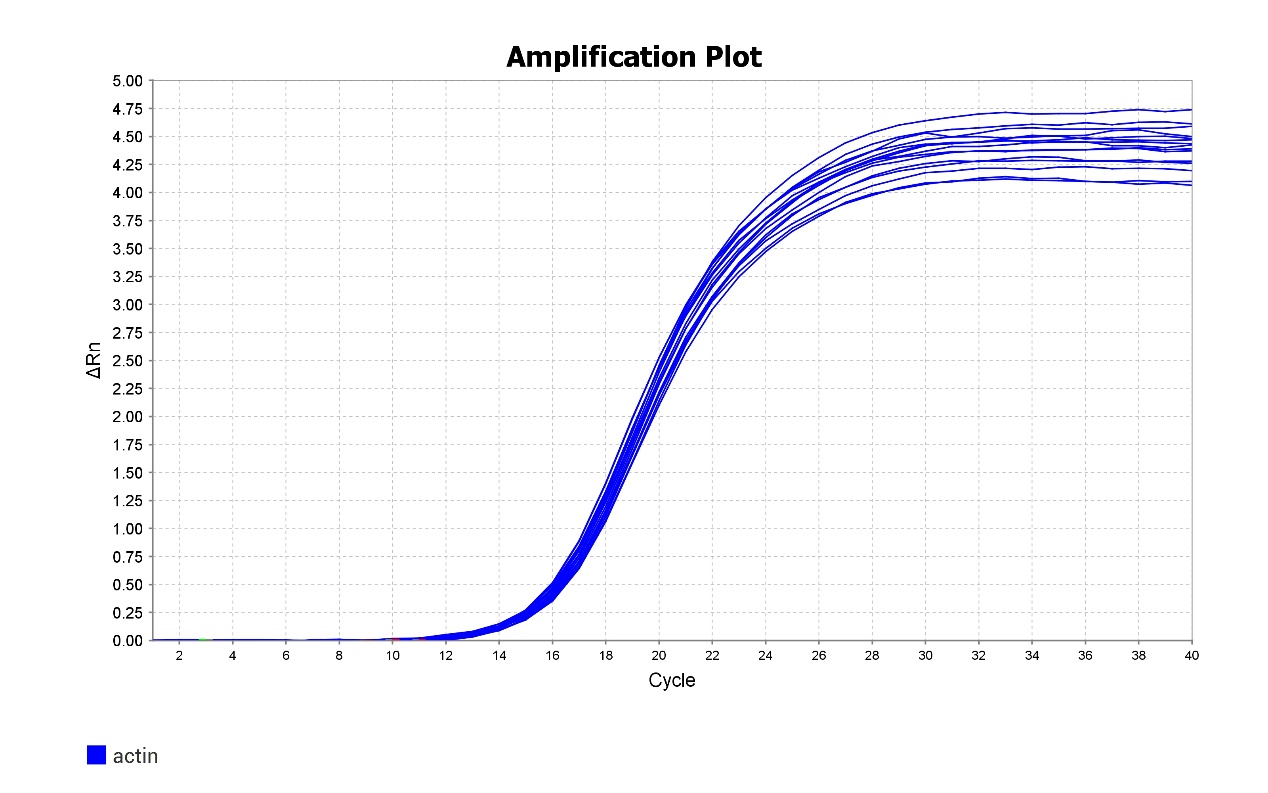


1-actin-Amplification Plot


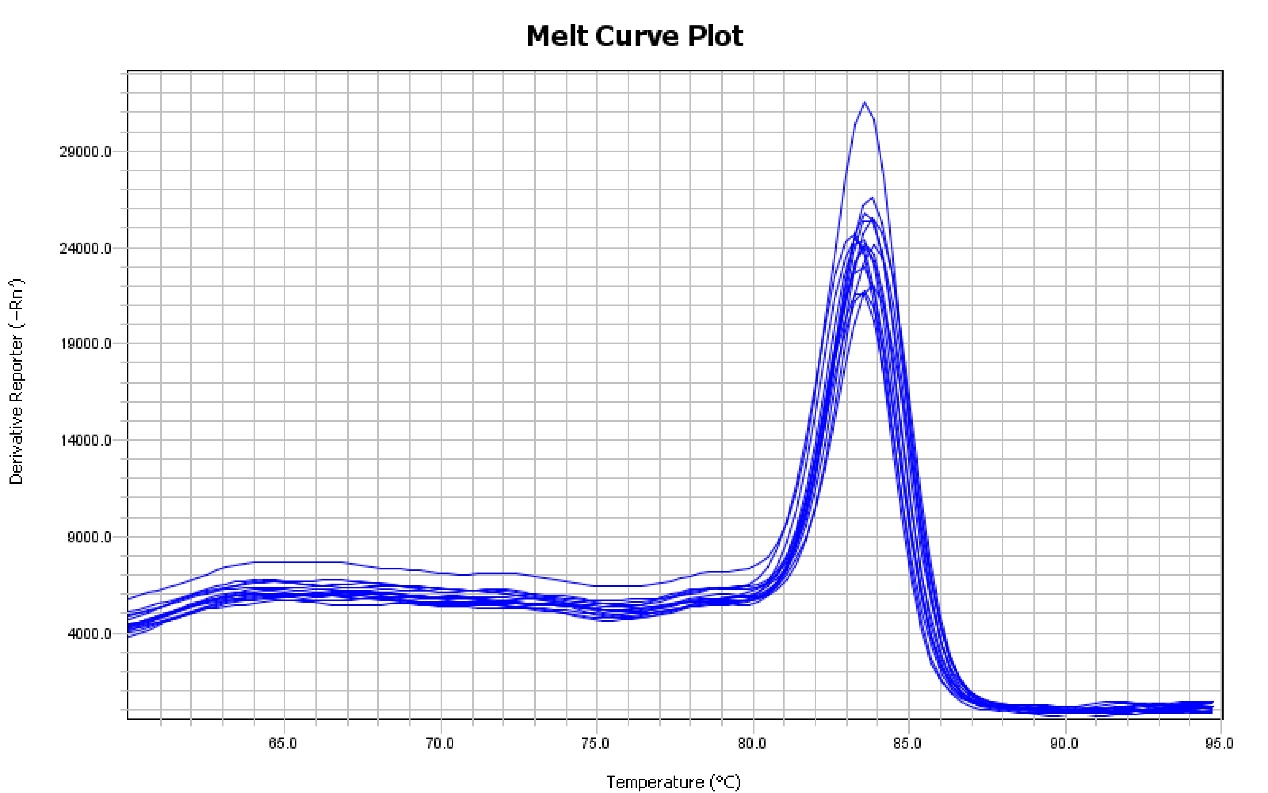


1-actin-Melt Curve Plot


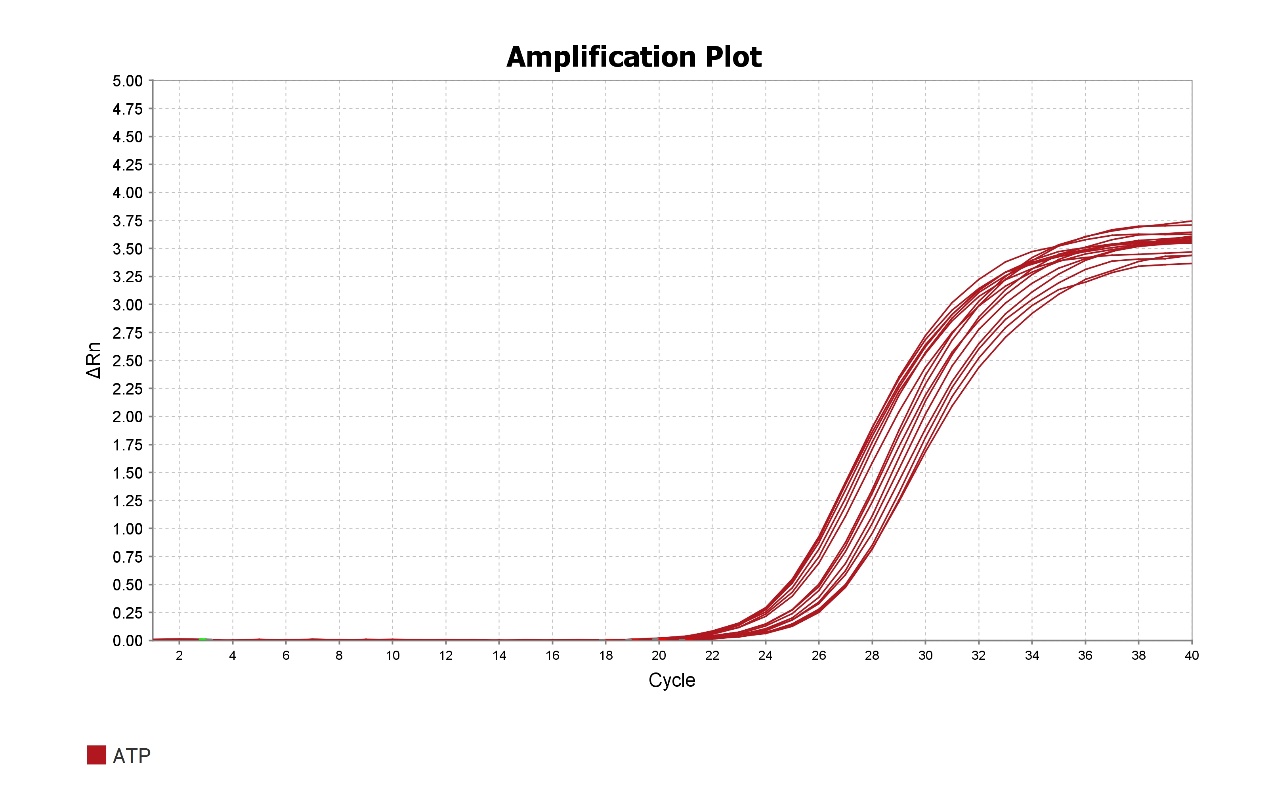


1-CEBPa-Amplification Plot


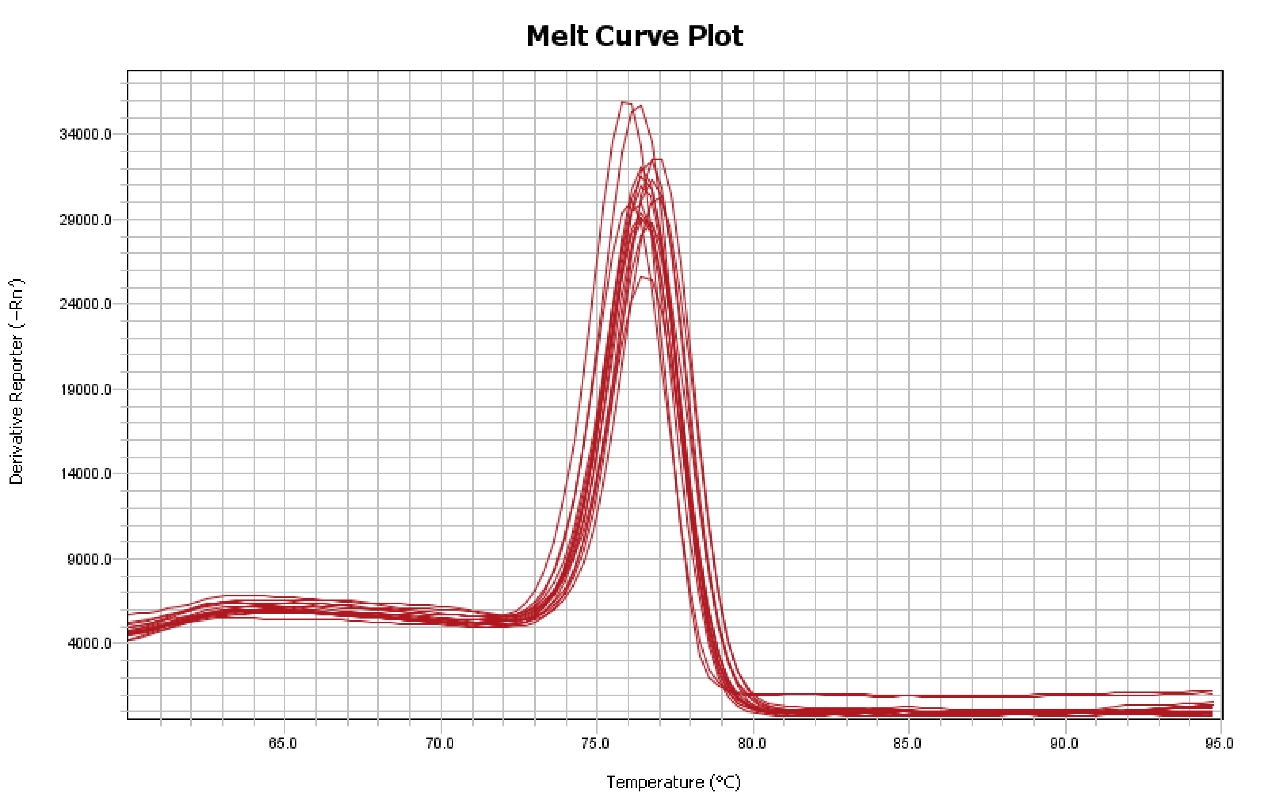


1-CEBPa-Melt Curve Plot
