# Supplementary figures and images for: Integrative genomic analysis reveals DHX58 as a key player in gastric cancer
Source: PLoS One. 2026 Jan 22;21(1):e0341230. doi: 10.1371/journal.pone.0341230 (PMC12826466; doi:10.1371/journal.pone.0341230)

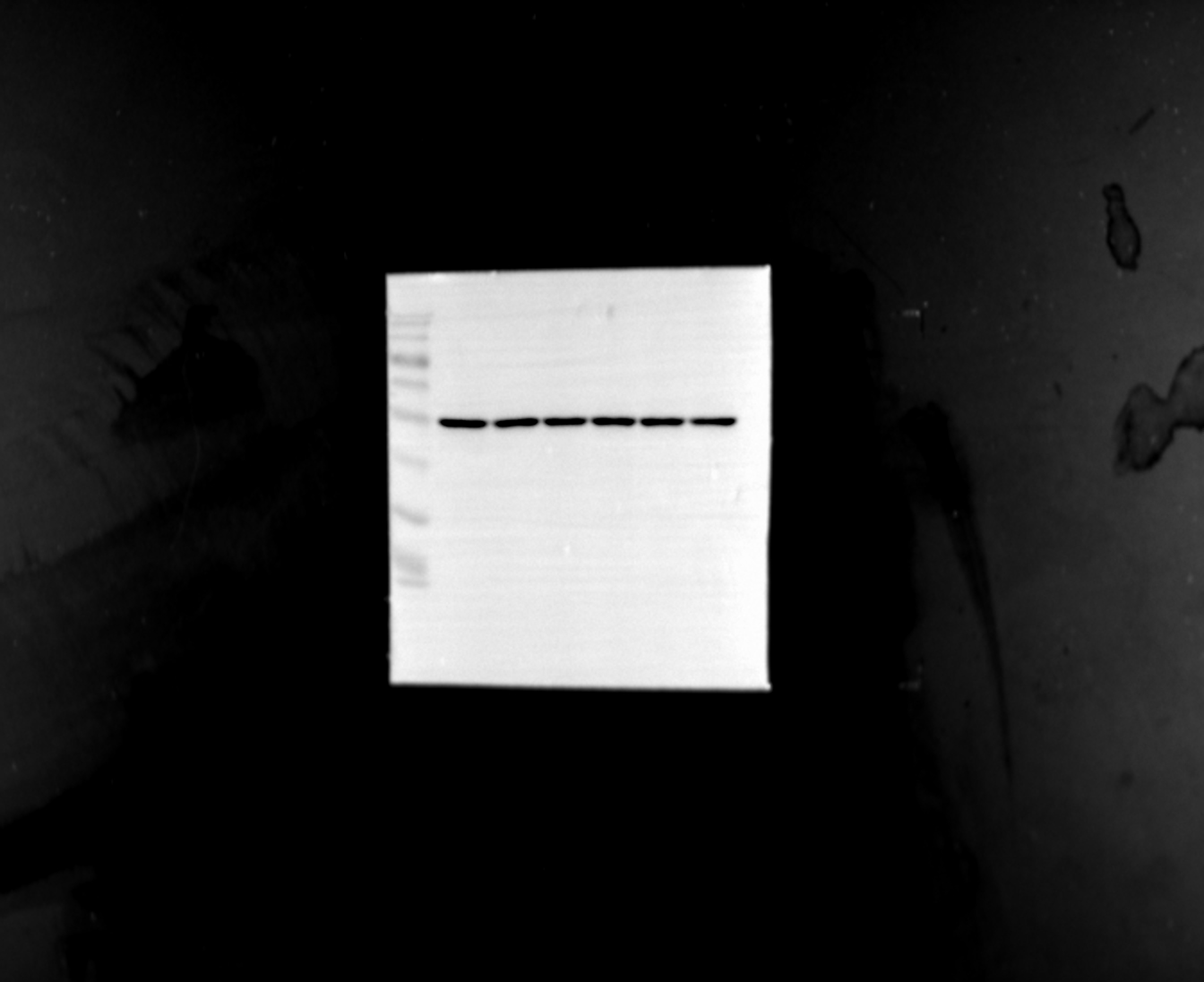

Supplement: S2 File — (TIF) [file pone.0341230.s002.tif]

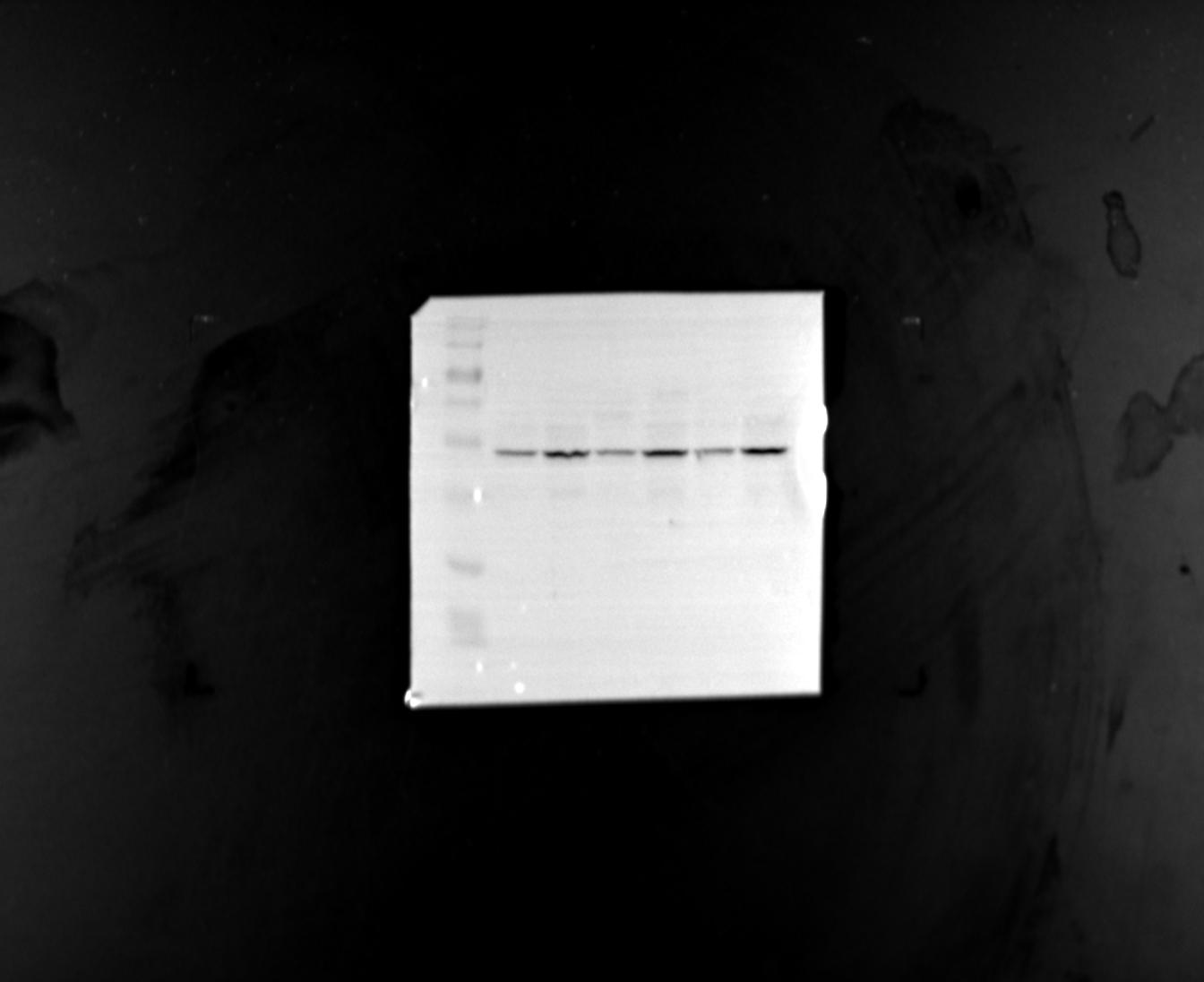

Supplement: S3 File — (TIF) [file pone.0341230.s003.tif]

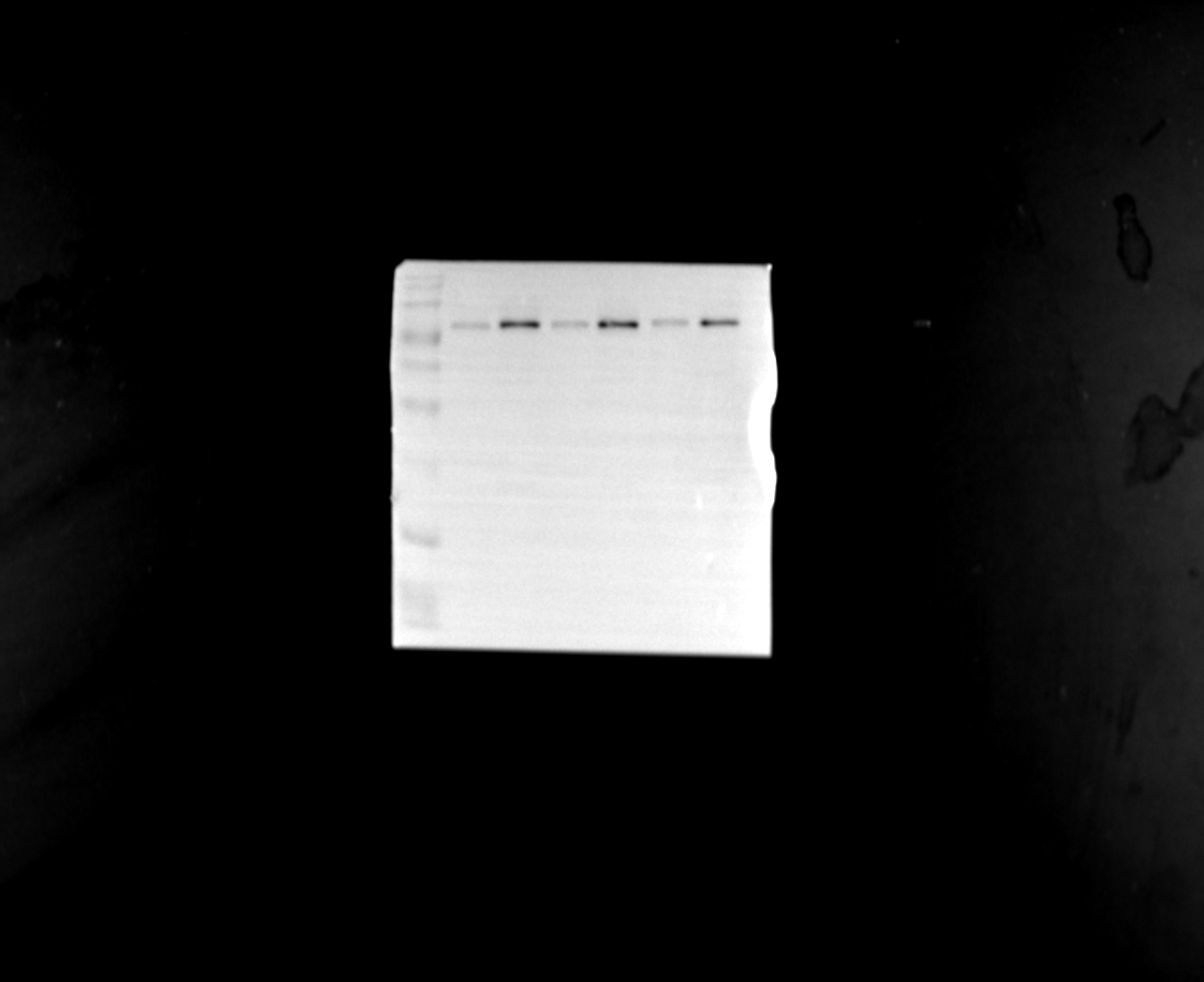

Supplement: S4 File — (TIF) [file pone.0341230.s004.tif]

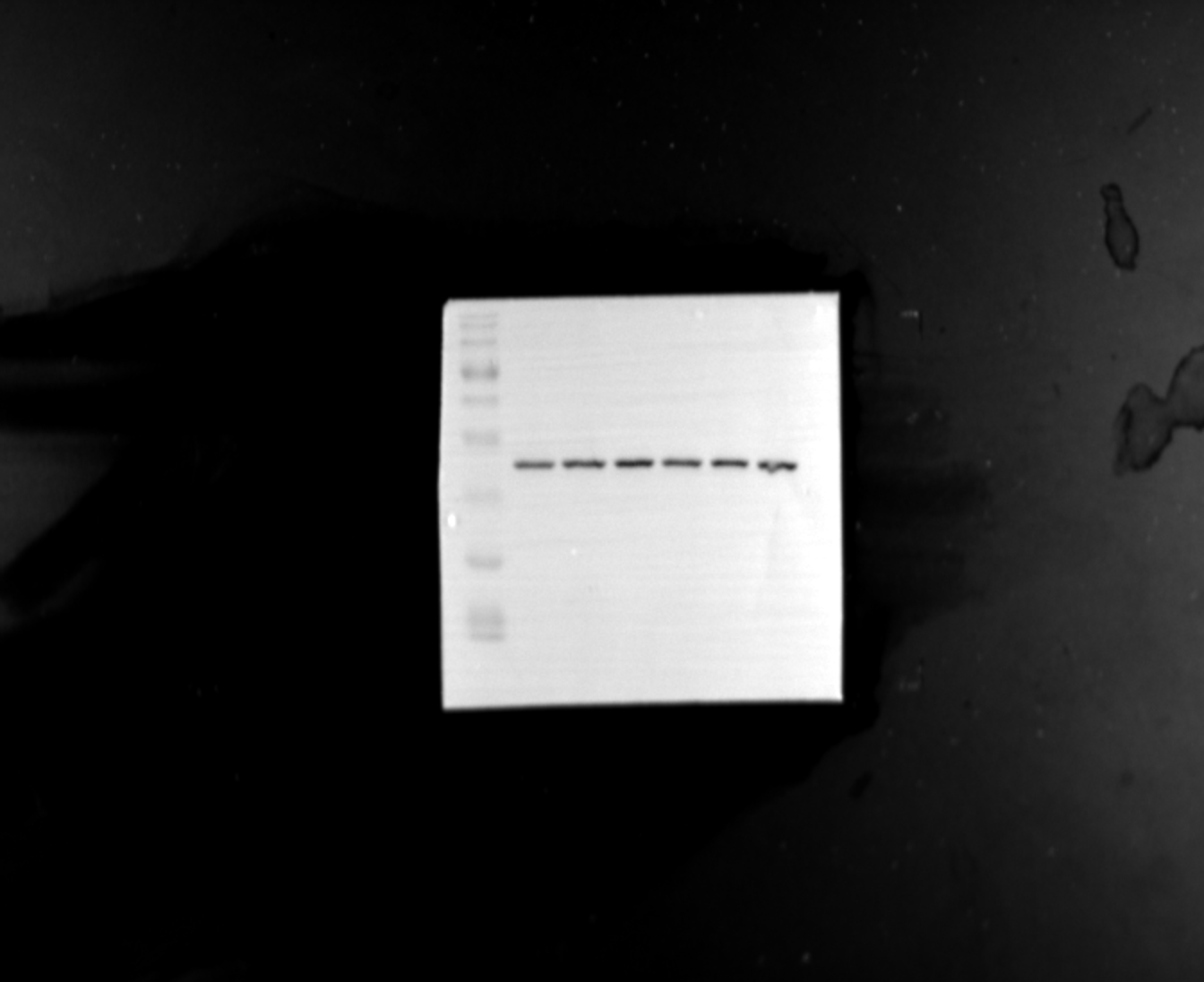

Supplement: S5 File — (TIF) [file pone.0341230.s005.tif]
